# Validating ECG-Based Artificial Intelligence Models for Cardiomyopathy Detection in Obstetric Patients: A Multisite International Study

**DOI:** 10.1016/j.jacadv.2026.103321

**Published:** 2026-09-22

**Authors:** Demilade Adedinsewo, Patrick W. Johnson, Carolina Morales-Lara, Maria Vitoria Coutinho, Sabrina D. Phillips, Marc A. Eidelhoch, Michal Cohen-Shelly, Elad Maor, Ariel Furer, Abraham Tsur, Shali Mazaki-Tovi, Kamilu M. Karaye, Bosede Bukola Afolabi, Olusoji Edward Jagun, Sadiq Hassan Ringim, Kehinde Femi Ibiyemi, Okechukwu Samuel Ogah, Sabha Bhatti, Shakeela Naz, Haleema Yasmin, Maedeh Ganji, Jose Ruiz, Khadeeja Esmail, Jason Wheatley, Anekwe Onwuanyi, Eric Chang, Myah M. Griffin, Oludamilola Oluleye, Stefanie Gefroh, Mohan Krishna Ranganathan, Samir Awasthi, Yvonne Butler Tobah, Francisco Lopez-Jimenez, Zachi I. Attia, Paul A. Friedman, Peter A. Noseworthy, Rickey E. Carter

**Affiliations:** a Department of Cardiovascular Medicine, Mayo Clinic, Jacksonville, Florida, USA; b Department of Quantitative Health Sciences, Mayo Clinic, Jacksonville, Florida, USA; c Department of Radiology, Emory University School of Medicine, Atlanta, Georgia, USA; d Leviev Heart Center, Sheba Medical Center, Ramat Gan, Israel; e ARC Innovation Center, Sagol AI Hub, Sheba Medical Center, Ramat Gan, Israel; f Gray Faculty of Medical and Health Sciences, Tel Aviv University, Ramat Aviv, Tel Aviv, Israel; g Department of Military Medicine, Faculty of Medicine, The Hebrew University of Jerusalem, Jerusalem, Israel; h Department of Obstetrics and Gynecology, The Sheba Medical Center, Tel Hashomer, Israel; i The Dina Recanati School of Medicine, Reichman University, Herzliya, Israel; j Department of Medicine, Aminu Kanu Teaching Hospital and Bayero University, Kano, Nigeria; k Department of Obstetrics and Gynecology, College of Medicine, University of Lagos, Idi-Araba, Lagos, Nigeria; l Centre for Clinical Trials, Research and Implementation Science, University of Lagos College of Medicine, Idi-Araba, Lagos, Nigeria; m Department of Obstetrics and Gynecology, Olabisi Onabanjo University Teaching Hospital, Sagamu, Ogun, Nigeria; n Department of Medicine, Rasheed Shekoni Federal University Teaching Hospital, Dutse, Jigawa, Nigeria; o Department of Obstetrics and Gynecology, University of Ilorin Teaching Hospital, Ilorin, Nigeria; p Cardiology Unit, Department of Medicine, University of Ibadan, Ibadan, Oyo, Nigeria; q Department of Adult Cardiology, National Institute of Cardiovascular Diseases (NICVD), Karachi, Pakistan; r Department of Cardiology, Rutgers Robert Wood Johnson University Hospital, New Brunswick, New Jersey, USA; s Department of Obstetrics and Gynecology, Jinnah Postgraduate Medical Centre (JPMC), Jinnah Sindh Medical University, Karachi, Pakistan; t Baptist Beaches Cardiology Center, Baptist Health, Jacksonville, Florida, USA; u Department of Transplant, Mayo Clinic, Jacksonville, Florida, USA; v Department of Cardiology, University of Florida College of Medicine, Jacksonville, Florida, USA; w Department of Obstetrics and Gynecology, University of Florida College of Medicine, Jacksonville, Florida, USA; x Department of Cardiology, Morehouse School of Medicine, Atlanta, Georgia, USA; y Department of Cardiology, West Georgia Cardiology, Carrollton, Georgia, USA; z Department of Obstetrics and Gynecology, Morehouse School of Medicine, Atlanta, Georgia, USA; aa Center for Maternal Health Equity, Department of Community Health and Preventive Medicine, Morehouse School of Medicine, Atlanta, Georgia, USA; ab Department of Cardiology, Essentia Health Heart and Vascular Center, Fargo, North Dakota, USA; ac Department of Obstetrics and Gynecology, Essentia Health, Fargo, North Dakota, USA; ad Anumana Inc, Cambridge, Massachusetts, USA; ae Nference Inc, Cambridge, Massachusetts, USA; af Department of Obstetrics and Gynecology, Mayo Clinic, Rochester, Minnesota, USA; ag Department of Cardiology, Mayo Clinic, Rochester, Minnesota, USA

**Keywords:** cardiomyopathies, ECG, heart failure, obstetrics, pregnancy, postpartum

## Abstract

**Background:**

Cardiomyopathy significantly impacts maternal health worldwide. Artificial intelligence–enabled electrocardiograms (AI-ECG) have shown effectiveness in detecting left ventricular systolic dysfunction (LVSD) in adults and limited obstetric cohorts; however, broad validation in diverse obstetric populations remains unexplored.

**Objectives:**

Our primary objective was to validate the performance of 2 versions of an AI-ECG model for detecting cardiomyopathy in an international obstetric cohort.

**Methods:**

We conducted a retrospective, multisite study of pregnant or postpartum women (≤12 months) across 11 institutions in 4 countries (Israel, Nigeria, Pakistan, and the United States). Two AI-ECG models (Food and Drug Administration–cleared v2.5.0 and refined v3.0.0) were evaluated against echocardiography. LVSD was defined as left ventricular ejection fraction ≤40% in keeping with the model’s training target. Model performance was assessed by area under the receiver-operating characteristic curve (AUROC).

**Results:**

We included 1,820 women with paired ECG-echocardiogram data, of whom 7.3% had LVSD. Model 1 (v2.5.0) demonstrated strong discriminative performance with an AUROC of 0.865, sensitivity of 46.1%, and negative predictive value of 95.8%. Model 2 (v3.0.0) showed significantly improved performance, with an AUROC of 0.925, sensitivity of 64.1%, and negative predictive value of 97.2% (*P* < 0.001) for LVSD detection.

**Conclusions:**

AI-ECG models demonstrated strong external validity in a global obstetric cohort, with significantly improved performance in the refined model trained on expanded data. These findings support the potential role of AI-ECG for a scalable LVSD screening tool in obstetric populations, while underscoring the importance of population-specific validation prior to clinical implementation.

Cardiomyopathy is a leading cause of morbidity and mortality among pregnant and postpartum women worldwide.^1^^,^^2^ The absence of routine screening for cardiomyopathy or left ventricular systolic dysfunction (LVSD) prior to conception and during the perinatal period limits our understanding of the true epidemiology of these conditions. As a result, it is believed that many patients with LVSD in the peripartum period, who are mildly symptomatic or asymptomatic, remain largely undiagnosed.^3^ Peripartum cardiomyopathy (PPCM) is a unique form of LVSD believed to occur towards the end of pregnancy or in the postpartum period,^4^ with global estimates reporting the highest incidence (1 in ∼100 livebirths) in Northern Nigeria.^5^ Similarly, data from the PEACE registry in Nigeria reported an LVSD prevalence (defined as left ventricular ejection fraction [LVEF] <50%) of 5.6% (6/108) among apparently healthy women during pregnancy and this increased to 10.2% (9/108) when followed at 6 to 8 weeks postpartum.^6^ Emerging data from the PRESAP (Pakistan Registry of Echocardiographic Screening in Asymptomatic Pregnant Women) suggests that the prevalence of LVSD in pregnancy may be higher than previously recognized. In a large cohort of asymptomatic pregnant women (n = 15,068) without a prior diagnosis of cardiovascular disease (CVD), PRESAP reported a prevalence of 2.2% for women with a LVEF <55% and approximately 1% for those with LVEF <40%.^7^

There is evidence that heart failure symptoms alone during pregnancy are poor discriminators of the presence of cardiomyopathy or LVSD^8^, ^9^, ^10^, ^11^ given a large proportion of pregnant women without heart disease may report similar symptoms. Data from the European Society of Cardiology EURObservational Research Programme registry also noted that a quarter of patients reporting mild symptoms had severe LVSD, with a LVEF ≤25%.^12^

Artificial intelligence–enabled electrocardiograms (AI-ECG) have been established as effective tools for the detection of LVSD, and this has been demonstrated in various patient populations and care settings.^13^, ^14^, ^15^, ^16^, ^17^ In obstetric patient populations, the AI-ECG has shown effectiveness in identifying LVSD in a retrospective study with an area under the receiver operating characteristic curve (AUROC) of 0.92 for LVEF ≤35%,^18^ a prospective pilot study (AUROC = 1.0 for LVEF <40%),^19^ and in a randomized controlled clinical trial of approximately 1,200 women in Nigeria (AUROC = 0.928 for LVEF <40%).^20^ These prior studies provide compelling evidence supporting the utility of AI tools for cardiomyopathy detection in obstetric care. However, variations in the sensitivity and positive predictive value (PPV) of the AI-ECG model have been observed, particularly in younger patient populations.^17^^,^^19^, ^20^, ^21^ Furthermore, large-scale external validation of the AI-ECG model across diverse obstetric patient populations and utilizing various 12-lead ECG devices had not been conducted. To address this gap, the objective of this study was to externally validate the diagnostic performance of the AI-ECG in detecting pregnancy-related cardiomyopathy within a diverse, international obstetric patient cohort. Additionally, the study aimed to evaluate the differences in diagnostic performance metrics among the existing versions of the AI-ECG model. We hypothesized that the refined version of the AI-ECG model would demonstrate improved discriminative performance for detecting pregnancy-related cardiomyopathy in an external obstetric cohort, compared with the earlier version, using 2-dimensional (2D) echocardiography as the reference standard.

## Methods

### Study design

This investigation was designed as a retrospective observational study. We obtained raw digital 12-lead ECG files in Extensible Markup Language (XML) or NumPy format from health care institutions located in 4 countries: Israel, Nigeria, Pakistan, and the United States. Data were obtained from 3 U.S. health care institutions (University of Florida, Jacksonville, FL; Grady Memorial Hospital, Atlanta, GA; and Essentia Heart and Vascular Center, Fargo, ND), 1 institution in Israel (Sheba Medical Center), 6 health care institutions in Nigeria participating in the SPEC-AI (NCT05438576) Nigeria^20^ study (Aminu Kano Teaching Hospital, Lagos University Teaching Hospital, Olabisi Onabanjo University Teaching Hospital, Rasheed Shekoni Specialist Hospital, University College Hospital Ibadan, and University of Ilorin Teaching Hospital), and 1 institution in Pakistan (National Institute of Cardiovascular Diseases, Karachi).

The study was approved by the Mayo Clinic Institutional Review Board as well as at all the participating sites. Informed consent was waived for this study, which utilized retrospective data available from the electronic health record (EHR) or collected as part of a previous research study. The Standards for Reporting of Diagnostic Accuracy Studies (STARD-AI) 2025 guidelines^22^^,^^23^ were utilized in the reporting of this study, which includes new and modified items in addition to the STARD 2015 checklist.^24^

### Study population

All available consecutive digital ECGs and paired echocardiogram reports were systematically extracted from the EHR across all participating sites in the United States and Israel. Data collection included ECGs and echocardiograms acquired through December 2024. Only pairs of ECGs and echocardiograms taken within a 30-day window of each other were included, as such individuals lacking the requisite ECG or echocardiographic data were not evaluated for study inclusion. The study focused exclusively on women of reproductive age (18-53 years), who were either pregnant at the time of the examination or within 12 months postpartum. Data from Nigeria and Pakistan were extracted from the SPEC-AI Nigeria^20^ (2022-2024) and PRESAP^7^ (2023-2025) studies, respectively. Research data use agreements were established between Mayo Clinic and all participating sites, and all study data were extracted between 2021 and 2025.

Standard 12-lead ECGs were acquired at each site at a sampling rate of 500 Hz using GE Healthcare, Philips Healthcare, and Bionet Medical ECG machines. ECGs obtained at a different sampling rate were excluded during data quality checks. The digital ECG waveforms were extracted from the 12-lead ECG device or from each site's ECG database management system in XML format. ECG files from Sheba Medical Center were first converted to a NumPy file prior to transfer to Mayo Clinic for analysis. In addition to the ECG files, additional metadata were extracted, when available, for each patient which included ECG device type (vendor, model, and software version), LVEF (based on 2D echocardiography), ECG timing in proximity to the index echocardiogram and index delivery date, and race/ethnicity. All echocardiograms were performed, and results were reported according to standard clinical and research procedures at each participating site. LVEF was estimated using either the biplane method of disks, 2D linear, M-mode, or visual assessment.

### Model descriptions

#### Model 1 (U.S. Food and Drug Administration cleared version 2.5.0, K250652)

Model 1 (v2.5.0) was a proprietary, U.S. Food and Drug Administration cleared, 12-lead ECG neural network for detection of LVSD (defined as LVEF ≤40% in alignment with the universal definition and classification of heart failure with reduced ejection fraction^25^), initially developed using paired ECG and echocardiogram data from approximately 94,000 patients seen at Mayo Clinic between January 1994 and February 2017. The average age of patients in the training data was 61.5 years,^26^ and this was externally validated in a cohort of 16,000 patients from 4 health systems in the United States with an average age of 66 years.^17^^,^^21^^,^^27^ For each ECG, the AI-ECG model generates a continuous score between 0 and 1 for the presence of LVSD. A prespecified threshold of 0.55995, established during model development as the optimal operating cutoff, was used to classify ECGs as positive or negative for LVSD. This version of the model incorporates cybersecurity enhancements, expanded compatibility with Philips PageWriter TC30 and TC50 devices, and an integrated quality control (QC) and preprocessing step for digital ECG files. This algorithm (Model 1) was specifically developed for use by clinicians and received approval from the US Food and Drug Administration as a cardiovascular machine learning–based notification software based in part on the performance estimates obtained from a multicenter external validation study.^17^ The primary purpose of this algorithm is to support the early detection of LVEF ≤40% in adults at risk for heart failure. Importantly, the use of this tool is intended to complement, not replace, clinical judgment, and it should be applied in conjunction with the expertise and assessment of a clinician.

#### Model 2 (version 3.0.0, Predetermined Change Control Plan compliant)

Model 2 uses the same neural network architecture and design as Model 1 but was retrained with additional data from approximately 346,000 Mayo Clinic patients, which included approximately 38,000 women between the ages of 18 and 49 years. The full training cohort consisted of 2.88 million ECGs with paired echocardiogram data (performed within 14 days) from 676,579 patients seen at Mayo Clinic between January 1994 and December 2023, expanding the overall training data set. The average age of patients in the training cohort was 65.4 years, 46.6% were male and 53.4% were female. This model was also fine-tuned to maximize overall diagnostic performance. The predetermined optimal threshold for LVSD detection derived at the time of model development was 0.1078, and this was used to classify ECGs as positive or negative for LVSD using the refined model. Model 2 employs identical QC and preprocessing protocols as Model 1. All enhancements to the model were implemented in strict accordance with the parameters outlined in a preapproved Predetermined Change Control Plan.^27^

### Statistical analysis

Only ECGs meeting the following criteria were included in the final analysis: 1) patients aged 18 years or older at the time of the ECG; 2) the ECG was performed within ±30 days of the echocardiogram; and 3) the ECG was acquired either within 32 weeks prior to the delivery date or up to 12 months postdelivery. Some of the data received included multiple combinations of ECGs and echocardiograms and delivery dates. To ensure consistency and avoid bias in our analysis, we selected only one ECG-echocardiogram pair linked to a single index delivery for each patient with multiple pairs using the following systematic approach: pairs with the shortest interval between the ECG and echocardiogram received the highest priority, if multiple were identified for a specific individual, then the pair with the earliest ECG was chosen, if more than one pair still existed, then the pair associated with the earliest delivery date was selected, and finally, if a tie breaker was still needed, then the earliest echocardiogram date was selected.

The ECGs were analyzed retrospectively using both AI-ECG models described, developed by Anumana Inc. The results generated by the AI models were not available to the clinicians and technicians who performed or interpreted the ECG and echocardiogram examinations at the time the data were acquired.

Statistical analyses were conducted using R (v4.2.2).^28^ All tests were 2-sided, and a *P* value < 0.05 was considered statistically significant. AI-ECG Software-as-a-Medical-Device models were executed within containerized environments using Podman (v4.9.4) and Python (v3.11.19).

### Data standardization, ECG linkage, and preprocessing

Data extraction protocols varied across each participating site, as such data had to be harmonized across all sites. ECGs received were linked to clinical data by either extracting patient ID, ECG filename, or ECG date/time from the ECG data files and matching this with the clinical data received. Data received from Israel included only time differences between events rather than actual dates (eg, time from birth to ECG and time from ECG to delivery) due to country-specific data privacy laws. As such, to standardize the data set, all patients in this cohort were assigned a date of birth on 1900-01-01, then all other dates were generated based on the time differences. LVEF values were provided as whole numbers (eg, 60%), ranges (eg, 45% to 50%), or thresholds (eg, >70%). For ease of reporting, the median value was utilized when a range was reported (eg, 45% to 50% would be represented as 47.5%). In instances where a specific threshold value was reported, the limit of detection was applied (eg, >70% would be represented as 70%). For tabular data, continuous numeric variables were expressed as the median along with the interquartile range, while categorical variables were presented as counts and column percentages. Diagnostic performance metrics, including the AUROC, area under the precision-recall curve (AUPRC), accuracy, sensitivity, specificity, PPV, and negative predictive value (NPV), were computed for each AI-ECG model. The differences in AUROC values among the AI-ECG models were assessed with the DeLong test.

## Results

A total of 1,820 unique patients (from all 4 countries) with paired ECG-echocardiogram data were deemed eligible for inclusion in the final analysis (Figure 1). The median age of the cohort was 31 years (Q1: 26.5, Q3: 35.5). Race information was not available for 676 women, as these data were not automatically tracked at specific sites. Among the sample with available race and ethnicity information, 69.2% (792/1,144) identified as Black, 20.5% (234/1,144) as South Asian, 8% (91/1,144) as White, and 1.9% (15/1,144) as Hispanic (Table 1). The median LVEF for the total cohort was 60% (Q1: 55.8, Q3: 62.0). One hundred and thirty-two (7.3%) women had an LVEF ≤40%. The median interval from ECG to echocardiogram was 0 days. Notably, 79.5% (1,447/1820) of ECGs were obtained using GE devices, while 12.9% (234/1820) were acquired with a Bionet device (CardioTouch 3000), and 7.6% (139/1820) utilized a Philips device (PageWriter TC70).

**Figure 1:**
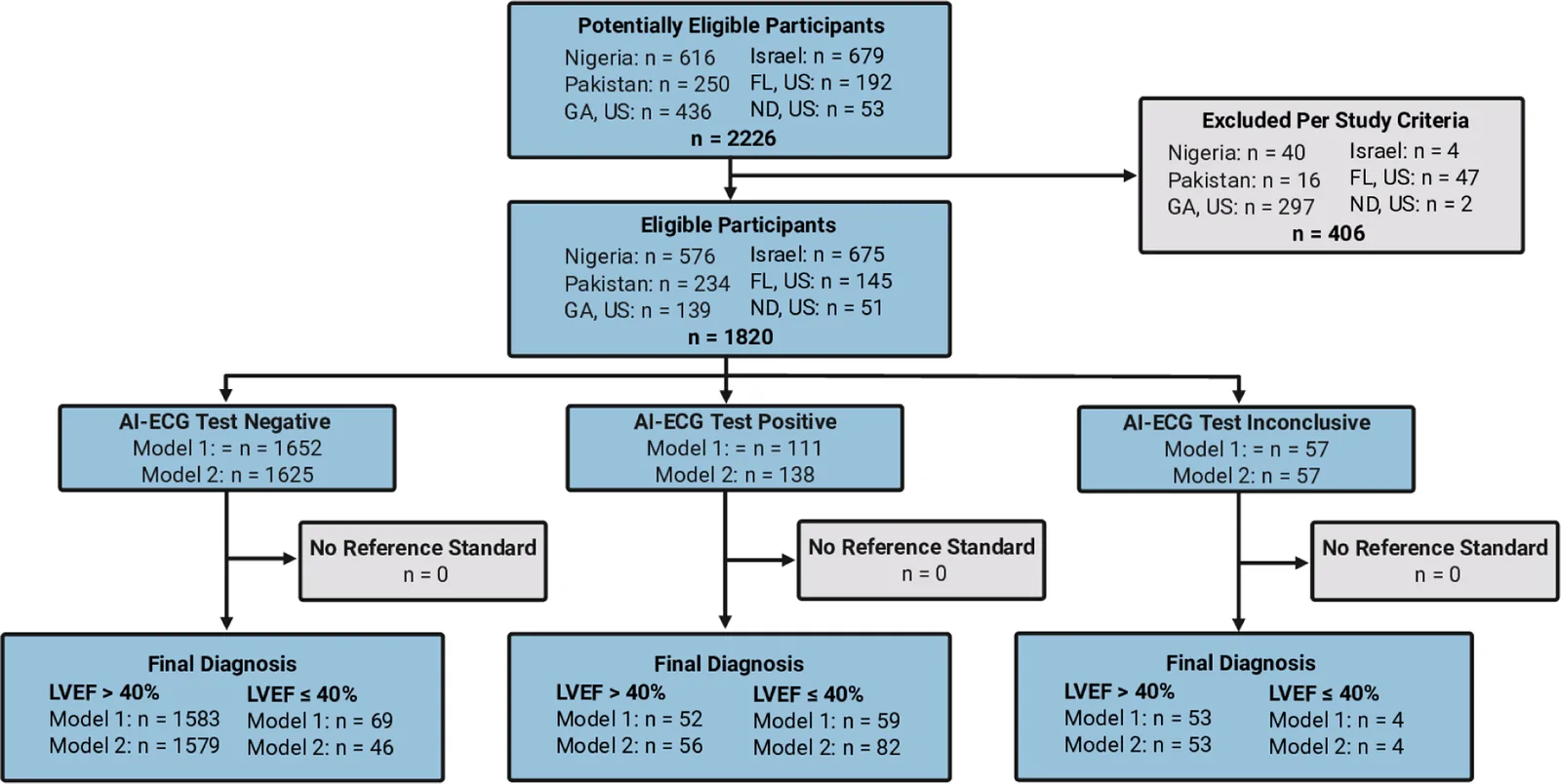
Flow of Participants in the Study Cohort Flow diagram illustrating patient selection, exclusions (due to missing or noneligible data), and inclusion in the final analytic cohort in accordance with STARD-AI guidelines. The diagram details the derivation of the external obstetric cohort and the final population used for external validation of the AI-ECG models for detection of pregnancy-related cardiomyopathy. LVEF = left ventricular ejection fraction.

**Table 1:** Demographics and Clinical Characteristics of the Study Sample

	GA, United States (n = 139)	Israel (n = 675)	Nigeria (n = 576)	ND, United States (n = 51)	Pakistan (n = 234)	FL, United States (n = 145)	Total (N = 1,820)
Age at ECG, y	28.0 (23.7, 34.8)	33.5 (29.6, 37.1)	30.9 (27.0, 35.5)	30.0 (26.8, 33.3)	25.8 (22.9, 29.9)	29.9 (25.1, 34.2)	31.0 (26.5, 35.5)
Race^a^							
Not reported	0	675	0	1	0	0	676
American Indian or Alaska Native	0 (0.0%)	0	0 (0.0%)	3 (6.0%)	0 (0.0%)	0 (0.0%)	3 (0.3%)
Asian	2 (1.4%)	0	0 (0.0%)	0 (0.0%)	0 (0.0%)	4 (2.8%)	6 (0.5%)
Black or African American	113 (81.3%)	0	576 (100.0%)	5 (10.0%)	0 (0.0%)	98 (67.6%)	792 (69.2%)
Hispanic	15 (10.8%)	0	0 (0.0%)	0 (0.0%)	0 (0.0%)	0 (0.0%)	15 (1.3%)
Multiracial	2 (1.4%)	0	0 (0.0%)	0 (0.0%)	0 (0.0%)	0 (0.0%)	2 (0.2%)
Native Hawaiian/Pacific Islander	0 (0.0%)	0	0 (0.0%)	1 (2.0%)	0 (0.0%)	0 (0.0%)	1 (0.1%)
South Asian	0 (0.0%)	0	0 (0.0%)	0 (0.0%)	234 (100.0%)	0 (0.0%)	234 (20.5%)
White or Caucasian	7 (5.0%)	0	0 (0.0%)	41 (82.0%)	0 (0.0%)	43 (29.7%)	91 (8.0%)
LVEF (%)	55.0 (50.0, 60.0)	60.0 (60.0, 60.0)	61.0 (56.5, 65.0)	57.5 (57.2, 63.0)	58.0 (58.0, 58.0)	50.0 (35.0, 57.5)	60.0 (55.8, 62.0)
LVEF ≤40%	24 (17.3%)	20 (3.0%)	20 (3.5%)	2 (3.9%)	18 (7.7%)	48 (33.1%)	132 (7.3%)
Time from ECG to ECHO (d)	0.0 (−2.5, 0.0)	0.0 (−2.0, 2.0)	0.0 (0.0, 0.0)	0.0 (0.0, 1.0)	0.0 (0.0, 0.0)	1.0 (0.0, 4.0)	0.0 (0.0, 0.0)
ECG completed before ECHO	87 (62.6%)	494 (73.2%)	576 (100.0%)	44 (86.3%)	233 (99.6%)	141 (97.2%)	1,575 (86.5%)
Time from ECG to delivery (d)	0.0 (−7.0, 29.5)	61.8 (6.9, 130.0)	33.2 (−1.8, 100.2)	NA	NA	0.0 (−10.0, 21.0)	35.2 (−1.8, 104.8)
ECG acquisition timing^a^							
Not specified	0	0	0	51	0	0	51
Postpartum	62 (44.6%)	134 (19.9%)	171 (29.7%)	0	0	72 (49.7%)	439 (24.8%)
During pregnancy	77 (55.4%)	541 (80.1%)	405 (70.3%)	0	234 (100%)	73 (50.3%)	1,330 (75.2%)
ECG device type							
Bionet CardioTouch	0	0	0	0	234 (100%)	0	234 (12.9%)
GE Healthcare	0 (0.0%)	675 (100.0%)	576 (100.0%)	51 (100.0%)	0	145 (100.0%)	1,447 (79.5%)
Philips Healthcare	139 (100.0%)	0 (0.0%)	0 (0.0%)	0 (0.0%)	0	0 (0.0%)	139 (7.6%)

Numeric data represented as median (q1, q3), categorical data as count (column percent).

Median, 25th percentile (Q1), and 75th percentile (Q3) reported for all numeric variables.

Frequency counts and percents reported for categorical variables.

ECHO = echocardiography; ECG = electrocardiogram; LVEF = left ventricular ejection fraction.

a Missing data were present and categorized as “not reported” or “not specified,” as such proportions were calculated based on available data excluding participants with missing information.

### Model performance

Fifty-seven ECGs did not have an AI-ECG output due to QC-related errors; 60% of these were due to the ECG voltage amplitude being outside of the accepted range. A final sample of 1763 unique patient ECGs was utilized for the AI predictions (2 × 2 analysis). To assess the potential impact of including lower-quality data on model performance, we performed an intention-to-screen analysis by incorporating all ECGs (n = 1820), including those failing QC checks. In this analysis, ECGs that failed QC were classified as either false negatives or false positives, as determined by the reference standard. This approach was used to evaluate a worst-case scenario for model performance (3 × 2 analysis). Model 1 had an AUROC of 0.865 (95% CI: 0.832-0.898) for detection of LVEF ≤40% (Figure 2) and achieved an AUPRC value of 0.478 in a cohort with 7.3% event prevalence, reflecting substantial improvement over chance performance. A 2 × 2 analysis (n = 1763) had a sensitivity of 46.1% (95% CI: 37.2%-55.1%) and a PPV of 53.2% (95% CI: 43.4%-62.7%). A 3 × 2 analysis (n = 1820) had a sensitivity of 44.7% (95% CI: 36.0%-53.6%) and a PPV of 36% (95% CI: 28.6%-43.8%). In contrast, Model 2 achieved an AUROC of 0.925 (95% CI: 0.901-0.949) for detection of LVEF ≤40% (Figure 3) and an AUPRC of 0.665, indicating meaningful precision-recall performance for identifying true positive cases. A 2 × 2 analysis (n = 1763) had a sensitivity of 64.1% (95% CI: 55.1%-72.3%) and a PPV of 59.4% (95% CI: 50.7%-67.7%). A 3 × 2 analysis (n = 1820) had a sensitivity of 62.1% (95% CI: 53.3%-70.4%) and a PPV of 42.9% (95% CI: 35.8%-50.3%). A comparison of both models demonstrated a statistically significant improvement in Model 2 compared to Model 1 (AUROC 0.925 vs 0.865) for detection of LVEF ≤40%, with a *P* value of <0.001. Figure 4 illustrates the agreement between echocardiography-derived binary LVEF categories (≤40% and >40%) and predictions from Model 1 and Model 2 using a Sankey plot, with flow width proportional to patient counts. Consistent with the observed AUROC performance, the Sankey plot demonstrates high agreement between model predictions and echocardiography-derived EF categories, with the majority of classifications aligning with the reference standard. The degree of agreement between Model 1 and Model 2 was 95.4% concordant in LVEF classification. However, Model 2 had a larger proportion of patients correctly classified as having an LVEF ≤40% visualized with the flow width. Figure 5 further quantifies intermodel agreement by showing the proportion of concordant classifications across clinically relevant LVEF categories (≤30%, 31% to 40%, 41% to 49%, and ≥50%), with Wilson 95% CIs illustrating the degree of uncertainty within each stratum. The highest intermodel agreement was observed in those with LVEF values ≥50%. Agreement was numerically lower across the LVEF <50% strata, with overlapping Wilson 95% CIs among the ≤30%, 31% to 40%, and 41% to 49% groups. It is important to note that agreement within strata reflects concordance between binary model classifications using the prespecified LVEF ≤40% threshold, rather than the accuracy of classification across the full range of LVEF values represented within each stratum.

**Figure 2:**
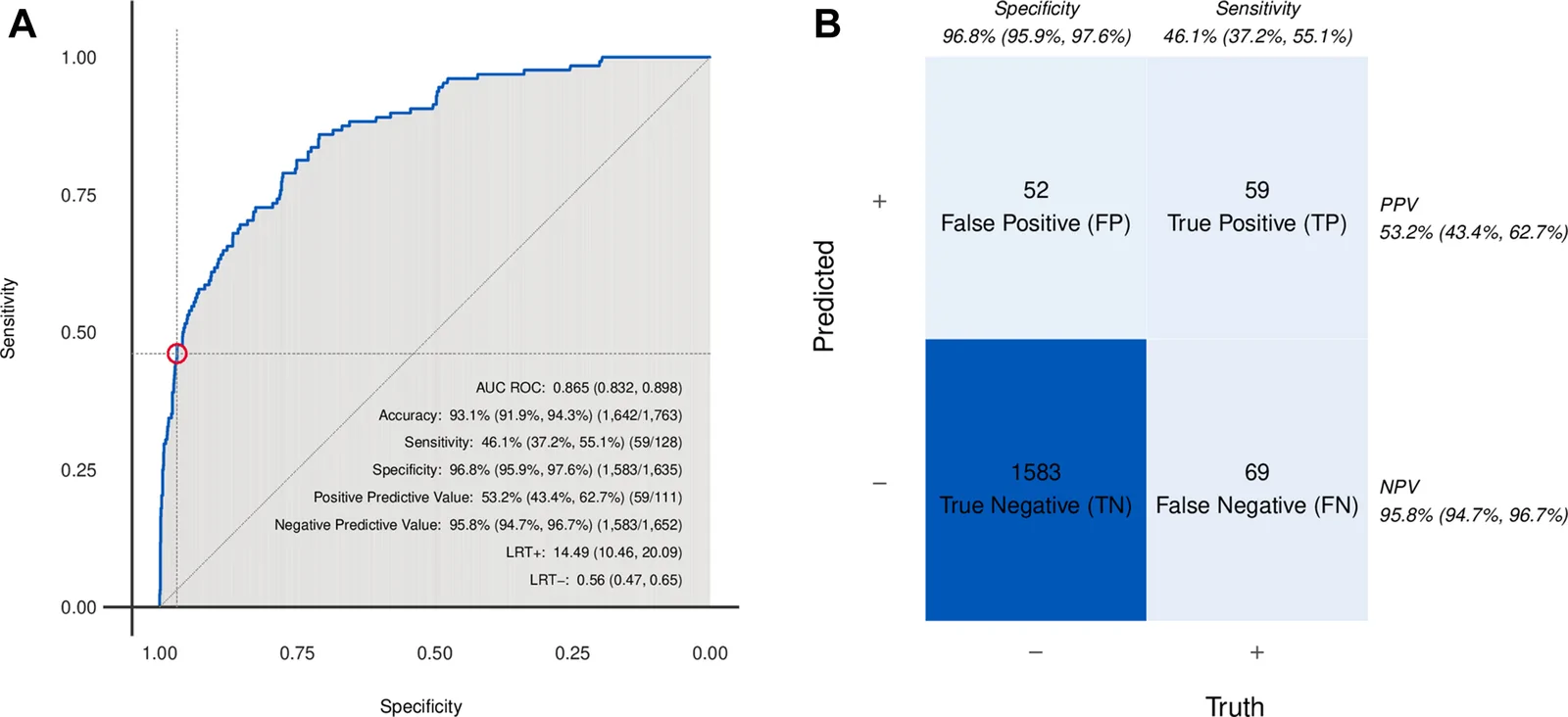
AI-ECG Model v2.5.0 (Model 1): Receiver-Operating Characteristic Curve and 2 × 2 Confusion Matrix for Detection of Left Ventricular Systolic Dysfunction A receiver-operating characteristic (ROC) curve (A) and corresponding 2 × 2 confusion matrix (B) summarizing the performance of the AI-ECG Model v2.5.0 for detecting left ventricular systolic dysfunction (LVSD; defined as LVEF ≤40%). The ROC curve displays the model’s discriminative ability across all model output thresholds, with the area under the curve (AUC) quantifying overall performance. The confusion matrix reports sensitivity, specificity, positive predictive value, and negative predictive value at the prespecified operating threshold (0.55995), illustrating classification outcomes relative to echocardiography-derived LVEF. LRT = likelihood ratio test; NPV = negative predictive value; PPV = positive predictive value; other abbreviation as in Figure 1.

**Figure 3:**
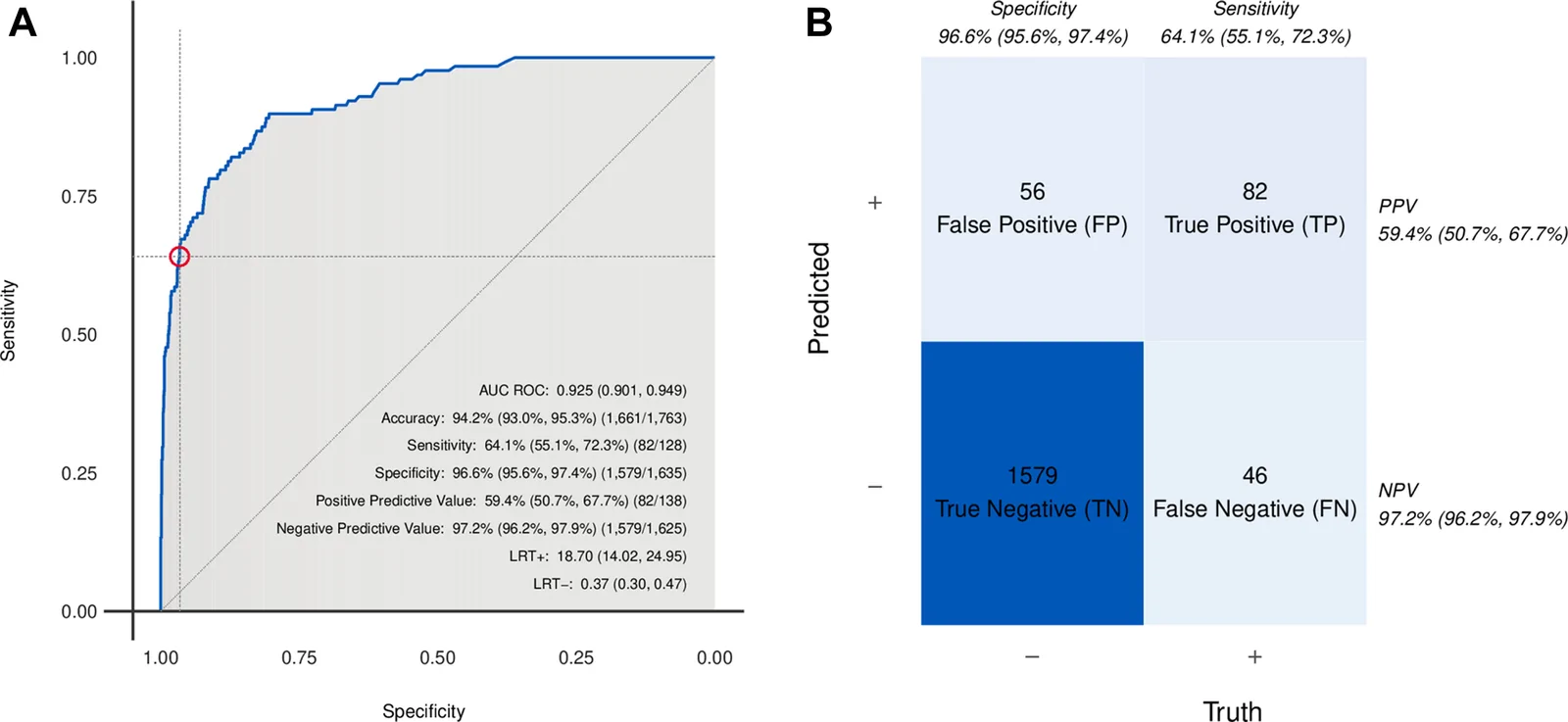
AI-ECG Model v3.0.0 (Model 2): Receiver-Operating Characteristic Curve and 2 × 2 Confusion Matrix for Detection of Left Ventricular Systolic Dysfunction A receiver-operating characteristic (ROC) curve (A) and corresponding 2 × 2 confusion matrix (B) summarizing the performance of the AI-ECG Model v3.0.0 for detecting left ventricular systolic dysfunction (LVSD; defined as LVEF ≤40%). The ROC curve displays the model’s discriminative ability across all model output thresholds, with the area under the curve (AUC) quantifying overall performance. The confusion matrix reports sensitivity, specificity, positive predictive value, and negative predictive value at the prespecified operating threshold (0.1078), illustrating classification outcomes relative to echocardiography-derived LVEF. Abbreviations as in Figures 1 and 2.

**Figure 4:**
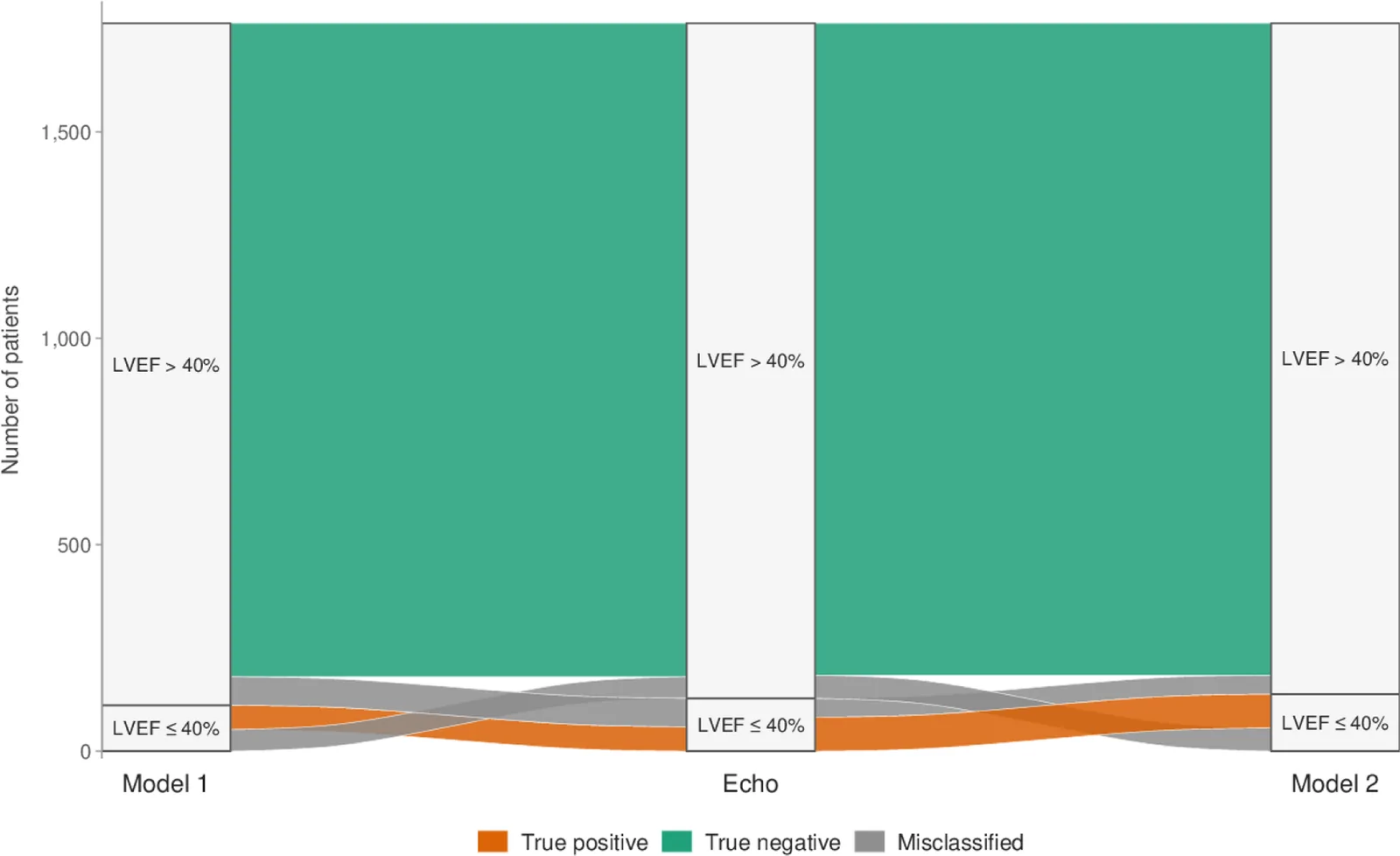
Sankey Diagram Showing Agreement Between Model 1 and Model 2 Across Binary Left Ventricular Ejection Fraction Categories A Sankey diagram demonstrating how patients transition across LVEF categories (LVEF >40% and LVEF ≤40%) from Model 1 (left) to echocardiography (middle) and Model 2 (right). Flow width represents the number of patients in each pathway, allowing visualization of concordant and discordant classifications between the 2 models relative to echocardiography. Abbreviation as in Figure 1.

**Figure 5:**
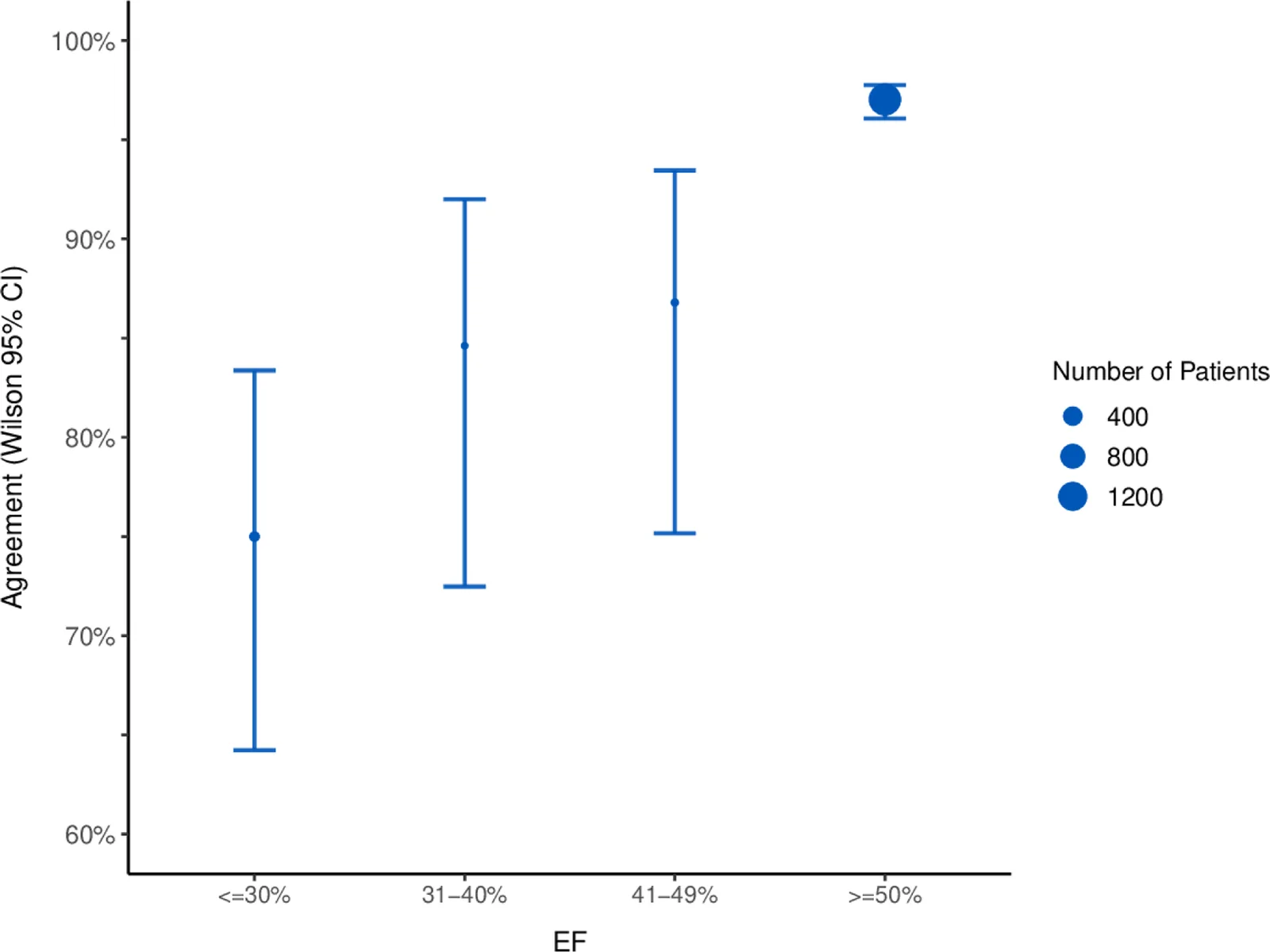
Intermodel Agreement Across Four Echocardiography-Defined LVEF Categories with 95% CIs A plot depicting the degree of agreement between Model 1 and Model 2 across echocardiography-defined LVEF categories (≤30%, 31% to 40%, 41% to 49%, and ≥50%). Point estimates represent the proportion of concordant classifications between the 2 models within each category, with vertical bars showing Wilson 95% CIs. Point size corresponds to the number of patients in each LVEF category. Abbreviation as in Figure 1.

**Central Illustration:**
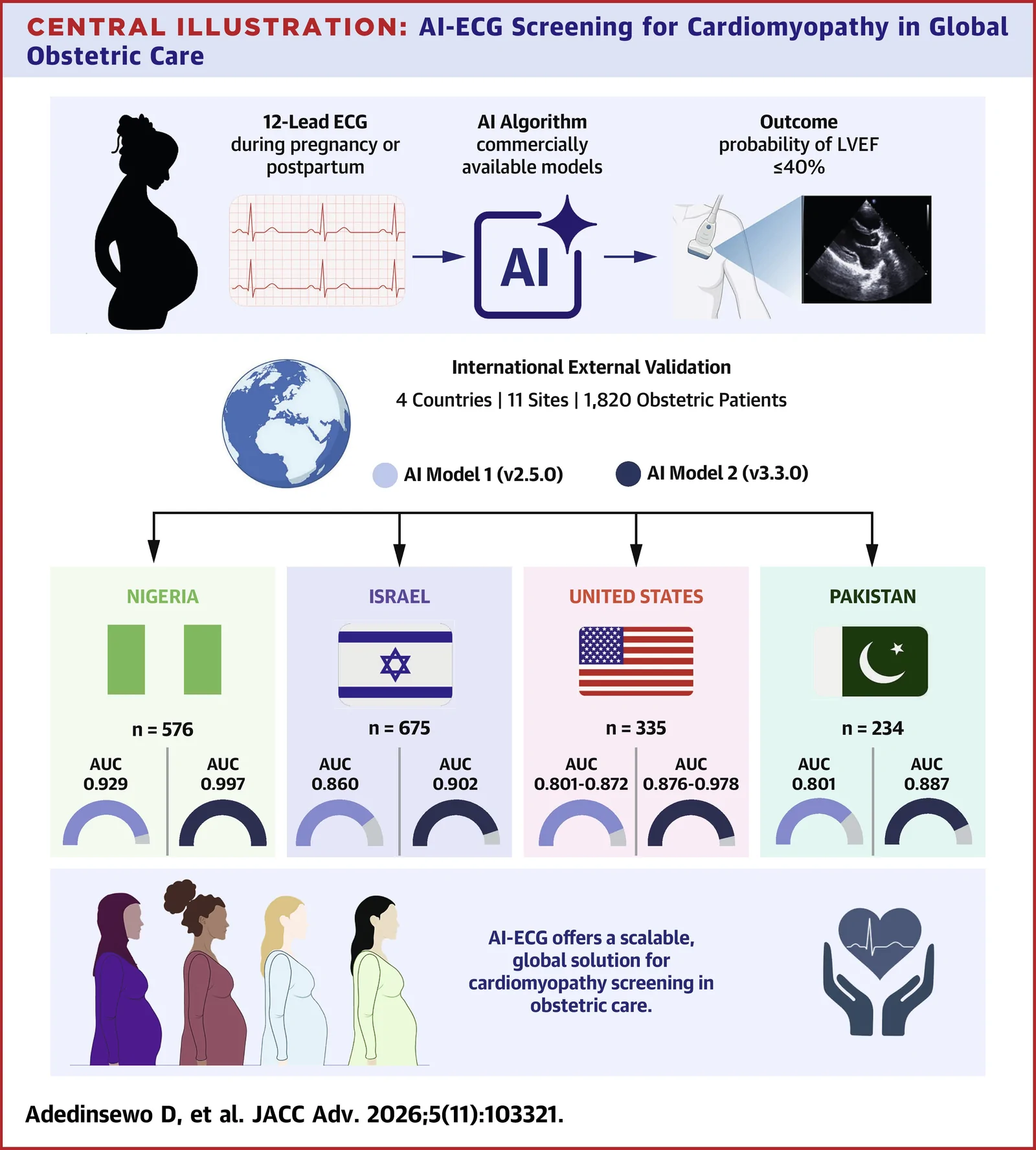
AI-ECG Screening for Cardiomyopathy in Global Obstetric Care This illustration depicts the application of artificial intelligence–enabled electrocardiography (AI-ECG) for detection of low left ventricular ejection fraction (LVEF) in pregnant and postpartum individuals in a multicenter international cohort. A standard 12-lead ECG obtained during the perinatal period is analyzed using 2 commercially available AI-ECG algorithms for detecting low LVEF. The models were externally validated in 1,820 women across 11 sites in four countries (United States, Nigeria, Israel, and Pakistan). The refined AI-ECG model demonstrated strong discriminative performance across all regions, albeit with site-level variations in sensitivity. Nevertheless, this study highlights the potential of AI-ECG as a scalable, noninvasive screening tool for early detection of cardiomyopathy in diverse obstetric populations globally. AUC = area under the curve.

For the detection of LVEF <45% (diagnostic cutoff for PPCM), using the prespecified binary classification thresholds with no additional training or modification, both models had similar diagnostic performance as shown with the detection of LVEF ≤40%. Specifically, Model 1 achieved an AUROC of 0.852 (95% CI: 0.817-0.887), while Model 2 achieved an AUROC of 0.909 (95% CI: 0.880-0.938). Complete diagnostic performance metrics are provided in Supplemental Table 1. Using the observed sensitivity and specificity, we estimated expected PPV, NPV, false positive and false negative counts across a range of hypothetical prevalence scenarios per 1,000 individuals screened. These estimates are provided in Supplemental Table 2. At a prevalence of 1%, the refined Model 2 (v3.0.0) would yield approximately 34 false positives and 4 false negatives per 1,000 individuals screened. This additional analysis was performed to provide clinicians with an illustrative estimate of the false positive burden associated with universal screening. Finally, to evaluate for potential geographic heterogeneity, we conducted a sensitivity analysis (Supplemental Table 3) excluding data from Pakistan because this site differed from the others in several key respects, including exclusive use of a Bionet ECG device, enrollment of an entirely South Asian cohort, and comparatively lower model sensitivity, suggesting possible threshold miscalibration in this population. Re-estimation of model performance after excluding the Pakistan cohort yielded AUROC values of 0.883 (95% CI: 0.852-0.915) for Model 1 and 0.937 (95% CI: 0.913-0.961) for Model 2, reflecting modest improvement without a substantial impact on overall conclusions.

### Subgroup analysis

We noticed variations in disease prevalence as well as the performance of the models by country and site, Tables 2 and 3. Model 2 performed best in all countries (Central Illustration). At the North American sites, LVSD detection performance metrics were as follows: in Georgia, AUROC was 0.876, sensitivity 75%, and PPV 52.9% (LVSD prevalence 17.3%, 24/139); in North Dakota, AUROC was 0.978, sensitivity 100%, and PPV 50% (LVSD prevalence 3.9%, 2/51); and in Florida, AUROC was 0.882, sensitivity 66.7%, and PPV 78% (LVSD prevalence 33.1%, 48/145).

**Table 2:** Diagnostic Performance Metrics for AI-ECG Model v2.5.0 (Model 1) Stratified by Key Variables of Interest

Group	Sensitivity	Specificity	AUC ROC	Positive Predictive Value	Negative Predictive Value
Site^a^					
Georgia (US)	50.0% (29.1%, 70.9%) (12/24)	90.3% (83.2%, 95.0%) (102/113)	0.801 (0.704, 0.898)	52.2% (30.6%, 73.2%) (12/23)	89.5% (82.3%, 94.4%) (102/114)
Israel	31.6% (12.6%, 56.6%) (6/19)	97.6% (96.0%, 98.6%) (603/618)	0.860 (0.768, 0.951)	28.6% (11.3%, 52.2%) (6/21)	97.9% (96.4%, 98.9%) (603/616)
Nigeria	76.5% (50.1%, 93.2%) (13/17)	97.3% (95.6%, 98.5%) (537/552)	0.929 (0.867, 0.991)	46.4% (27.5%, 66.1%) (13/28)	99.3% (98.1%, 99.8%) (537/541)
North Dakota (US)	50.0% (1.3%, 98.7%) (1/2)	93.5% (82.1%, 98.6%) (43/46)	0.859 (0.687, 1.000)	25.0% (0.6%, 80.6%) (1/4)	97.7% (88.0%, 99.9%) (43/44)
Pakistan	22.2% (6.4%, 47.6%) (4/18)	99.5% (97.4%, 100.0%) (208/209)	0.801 (0.700, 0.901)	80.0% (28.4%, 99.5%) (4/5)	93.7% (89.6%, 96.5%) (208/222)
Florida (US)	47.9% (33.3%, 62.8%) (23/48)	92.8% (85.7%, 97.0%) (90/97)	0.872 (0.813, 0.931)	76.7% (57.7%, 90.1%) (23/30)	78.3% (69.6%, 85.4%) (90/115)
Predelivery ECG^b^					
No	54.4% (41.9%, 66.5%) (37/68)	94.7% (91.8%, 96.8%) (338/357)	0.882 (0.838, 0.926)	66.1% (52.2%, 78.2%) (37/56)	91.6% (88.3%, 94.2%) (338/369)
Yes	42.5% (27.0%, 59.1%) (17/40)	97.2% (96.0%, 98.1%) (994/1,023)	0.871 (0.817, 0.925)	37.0% (23.2%, 52.5%) (17/46)	97.7% (96.6%, 98.6%) (994/1,017)
Unknown	25.0% (8.7%, 49.1%) (5/20)	98.4% (96.0%, 99.6%) (251/255)	0.802 (0.709, 0.895)	55.6% (21.2%, 86.3%) (5/9)	94.4% (90.9%, 96.8%) (251/266)
Maternal age group^c^					
<30 y	36.0% (22.9%, 50.8%) (18/50)	97.0% (95.5%, 98.2%) (690/711)	0.800 (0.736, 0.864)	46.2% (30.1%, 62.8%) (18/39)	95.6% (93.8%, 96.9%) (690/722)
30+ y	52.6% (40.9%, 64.0%) (41/78)	96.6% (95.3%, 97.7%) (893/924)	0.907 (0.876, 0.939)	56.9% (44.7%, 68.6%) (41/72)	96.0% (94.6%, 97.2%) (893/930)
Device vendor^d^					
Bionet	22.2% (6.4%, 47.6%) (4/18)	99.5% (97.4%, 100.0%) (208/209)	0.801 (0.700, 0.901)	80.0% (28.4%, 99.5%) (4/5)	93.7% (89.6%, 96.5%) (208/222)
GE Healthcare	50.0% (39.0%, 61.0%) (43/86)	97.0% (95.9%, 97.8%) (1,273/1,313)	0.889 (0.853, 0.925)	51.8% (40.6%, 62.9%) (43/83)	96.7% (95.6%, 97.6%) (1,273/1,316)
Philips Healthcare	50.0% (29.1%, 70.9%) (12/24)	90.3% (83.2%, 95.0%) (102/113)	0.801 (0.704, 0.898)	52.2% (30.6%, 73.2%) (12/23)	89.5% (82.3%, 94.4%) (102/114)
Overall	46.1% (37.2%, 55.1%) (59/128)	96.8% (95.9%, 97.6%) (1,583/1,635)	0.865 (0.832, 0.898)	53.2% (43.4%, 62.7%) (59/111)	95.8% (94.7%, 96.7%) (1,583/1,652)

AUC = area under the curve; ROC = receiver-operating characteristic; other abbreviation as in Table 1.

a Breslow-day tests, *P* = 0.01.

b Breslow-day tests *P* = 0.93.

c Breslow-day tests *P* = 0.24.

d Breslow-day tests *P* = 0.07.

**Table 3:** Diagnostic Performance Metrics for AI-ECG Model v3.0.0 (Model 2) Stratified by Key Variables of Interest

Group	Sensitivity	Specificity	AUC ROC	Positive Predictive Value	Negative Predictive Value
Site^a^					
Georgia (US)	75.0% (53.3%, 90.2%) (18/24)	85.8% (78.0%, 91.7%) (97/113)	0.876 (0.794, 0.959)	52.9% (35.1%, 70.2%) (18/34)	94.2% (87.8%, 97.8%) (97/103)
Israel	47.4% (24.4%, 71.1%) (9/19)	97.6% (96.0%, 98.6%) (603/618)	0.902 (0.831, 0.973)	37.5% (18.8%, 59.4%) (9/24)	98.4% (97.0%, 99.2%) (603/613)
Nigeria	100.0% (80.5%, 100.0%) (17/17)	97.5% (95.8%, 98.6%) (538/552)	0.997 (0.994, 1.000)	54.8% (36.0%, 72.7%) (17/31)	100.0% (99.3%, 100.0%) (538/538)
North Dakota (US)	100.0% (15.8%, 100.0%) (2/2)	95.7% (85.2%, 99.5%) (44/46)	0.978 (0.926, 1.000)	50.0% (6.8%, 93.2%) (2/4)	100.0% (92.0%, 100.0%) (44/44)
Pakistan	22.2% (6.4%, 47.6%) (4/18)	100.0% (98.3%, 100.0%) (209/209)	0.887 (0.814, 0.959)	100.0% (39.8%, 100.0%) (4/4)	93.7% (89.7%, 96.5%) (209/223)
Florida (US)	66.7% (51.6%, 79.6%) (32/48)	90.7% (83.1%, 95.7%) (88/97)	0.882 (0.825, 0.939)	78.0% (62.4%, 89.4%) (32/41)	84.6% (76.2%, 90.9%) (88/104)
Predelivery ECG^b^					
No	75.0% (63.0%, 84.7%) (51/68)	92.7% (89.5%, 95.2%) (331/357)	0.916 (0.875, 0.956)	66.2% (54.6%, 76.6%) (51/77)	95.1% (92.3%, 97.1%) (331/348)
Yes	62.5% (45.8%, 77.3%) (25/40)	97.3% (96.1%, 98.2%) (995/1,023)	0.944 (0.915, 0.973)	47.2% (33.3%, 61.4%) (25/53)	98.5% (97.6%, 99.2%) (995/1,010)
Unknown	30.0% (11.9%, 54.3%) (6/20)	99.2% (97.2%, 99.9%) (253/255)	0.885 (0.815, 0.955)	75.0% (34.9%, 96.8%) (6/8)	94.8% (91.4%, 97.1%) (253/267)
Maternal age group^c^					
<30 y	60.0% (45.2%, 73.6%) (30/50)	96.8% (95.2%, 97.9%) (688/711)	0.901 (0.855, 0.947)	56.6% (42.3%, 70.2%) (30/53)	97.2% (95.7%, 98.3%) (688/708)
30+ y	66.7% (55.1%, 76.9%) (52/78)	96.4% (95.0%, 97.5%) (891/924)	0.941 (0.917, 0.966)	61.2% (50.0%, 71.6%) (52/85)	97.2% (95.9%, 98.1%) (891/917)
Device vendor^d^					
Bionet	22.2% (6.4%, 47.6%) (4/18)	100.0% (98.3%, 100.0%) (209/209)	0.887 (0.814, 0.959)	100.0% (39.8%, 100.0%) (4/4)	93.7% (89.7%, 96.5%) (209/223)
GE Healthcare	69.8% (58.9%, 79.2%) (60/86)	97.0% (95.9%, 97.8%) (1,273/1,313)	0.944 (0.919, 0.969)	60.0% (49.7%, 69.7%) (60/100)	98.0% (97.1%, 98.7%) (1,273/1,299)
Philips Healthcare	75.0% (53.3%, 90.2%) (18/24)	85.8% (78.0%, 91.7%) (97/113)	0.876 (0.794, 0.959)	52.9% (35.1%, 70.2%) (18/34)	94.2% (87.8%, 97.8%) (97/103)
Overall	64.1% (55.1%, 72.3%) (82/128)	96.6% (95.6%, 97.4%) (1,579/1,635)	0.925 (0.901, 0.949)	59.4% (50.7%, 67.7%) (82/138)	97.2% (96.2%, 97.9%) (1,579/1,625)

Abbreviations as in Tables 1 and 2.

a Breslow-day tests, *P* < 0.01.

b Breslow-day tests, *P* = 0.67.

c Breslow-day tests, *P* = 0.69.

d Breslow-day tests, *P* = 0.04.

In Israel (LVSD prevalence was 3%, 20/675), Model 2 achieved an AUROC of 0.902, a sensitivity of 47.4%, and a PPV of 37.5%. In the Nigerian cohort (LVSD prevalence 3.5%, 20/576), Model 2 achieved an AUROC of 0.997, sensitivity of 100%, and PPV 54.8%. In Pakistan (LVSD prevalence 7.7%, 18/234), Model 2 achieved an AUROC of 0.887, a sensitivity of 22.2%, and a PPV of 100%.

### GE system 12-lead ECG

AI-ECG Model performance was highest with GE 12-lead ECGs (which were predominantly utilized across North American, Israeli, and Nigerian study sites). For Model 1, AUROC was 0.889 (95% CI: 0.853-0.925) with a sensitivity of 50%, and for Model 2, AUROC was 0.944 (95% CI: 0.919-0.969) and sensitivity was 69.8% (Tables 2 and 3).

### Phillips system 12-lead ECG

Only one site used the Philips ECG system, as such, Models 1 and 2 had the same performance metrics as that single site (Grady Memorial Hospital), demonstrating good performance overall with AUROC values of 0.801 and 0.876 (Tables 2 and 3).

### Bionet system 12-lead ECG

Model performance was notably lowest with the Bionet system 12-lead ECGs, which were utilized only in Pakistan. As such, Models 1 and 2 had the same performance metrics as the site in Pakistan (AUROC: 0.801 and 0.887). Sensitivity was particularly poor, 22.2% for both models at the preselected threshold despite an acceptable AUROC value (Tables 2 and 3).

## Discussion

We conducted an external validation of 2 AI-ECG models for LVSD detection in a large, geographically diverse, population of pregnant and postpartum women using retrospective data and demonstrated strong performance of the AI models (trained primarily on data from patients in the United States) across multiple countries. We also demonstrate the superior performance of the refined AI-ECG model (v3.0.0), which included additional training data, for detection of LVEF <40% (which the model was trained and optimized for) as well as LVEF <45% (diagnostic threshold for PPCM). However, we noted some variability in specific performance metrics across sites with the lowest sensitivity observed in the Pakistani cohort.

Several hypotheses may account for the lower sensitivity of the AI-ECG models in the Pakistani cohort. First, all ECGs were obtained using Bionet health care machines, and the AI-ECG models had limited prior exposure to data from this specific device. Second, inconsistencies were noted between ECG waveform measurements in the exported XML files and those displayed on printed PDF reports. As a result, raw digital waveform measurements required recalibration against the PDF values rather than relying solely on XML metadata. Third, LVEF values in this data set were exclusively from visual estimates and were reported as ranges, necessitating the use of median values for analysis, which may have contributed to errors in ascertainment of LVEF values. Finally, echocardiogram images were acquired using either standard transthoracic echocardiogram machines or handheld ultrasound devices, whereas all other sites employed standard transthoracic echocardiogram machines exclusively.

This study underscores the importance of validating AI models within the intended patient populations before clinical implementation, particularly in diverse clinical environments. Notably, while the AI models exhibited low sensitivity in the Pakistani cohort, their overall AUROC values remained robust, consistently exceeding 0.75 and reaching as high as 0.86, comparable to those reported in other AI-ECG studies.^29^^,^^30^ These findings highlight that, despite variability in sensitivity, the discriminative ability of the models was preserved across different populations. There might be a role for threshold calibration within unique patient populations, rather than an out-of-the-box cut-point value that aims to optimize model performance characteristics for the average population in which it was trained. Due to the absence of large ECG and echocardiogram databases among pregnant and postpartum individuals, all prior studies evaluating the AI-ECG in obstetric patients, including ours, utilized models that were previously trained with nonpregnant, as well as adult male patients. These training datasets included predominantly middle-aged patient populations (average age >50 years in the Tennessee heart failure cohort and >60 years in the South Korean and Mayo Clinic cohort).^26^^,^^30^^,^^31^ Despite the strong overall performance of both AI-ECG models in the current study, as indicated by the AUROC values, key diagnostic metrics such as sensitivity remained limited for both models, although Model 2 consistently outperformed Model 1. This finding suggests that the predetermined threshold, established based on the broader training cohort, may not be ideally suited for the unique population of pregnant and postpartum individuals. While applying a single threshold for LVSD detection across all patient populations is appealing for its simplicity, a uniform one-size-fits-all approach may not yield optimal results.^32^ Evidence from biomarker-based screening, such as high-sensitivity cardiac troponin, natriuretic peptides, and d-dimer, demonstrates that threshold values often require adjustment for specific patient groups (eg, by sex, cardiac rhythm, age, presence of renal dysfunction, or assay type).^25^^,^^33^, ^34^, ^35^ With high-sensitivity cardiac troponin, threshold values were selected to maximize NPV for ruling out myocardial infarction.^33^ However, studies have shown that applying uniform thresholds for both sexes may lead to reduced sensitivity and NPV in women, potentially contributing to sex bias in the diagnosis and management of acute coronary syndromes.^34^ Therefore, further validation may warrant the consideration of population-specific threshold values for AI-ECG prediction values in pregnant and postpartum patients, with the goal of improving sensitivity and minimizing missed cases of LVSD, particularly given the association between delayed diagnosis and adverse maternal and infant outcomes. It is important to note that the reported model performance metrics are based on a cohort of women who either exhibited signs or symptoms of CVD or for whom there was a high clinical suspicion of CVD. Therefore, model performance may differ in a general obstetric population with lower disease prevalence. This study also highlights the need to curate large digital ECG and echocardiogram databases among women of reproductive age, particularly during pregnancy and postpartum.

Our findings add to the growing body of literature evaluating the development and validation of ECG-based AI algorithms for LVSD detection in obstetric patients. Prior to the development of AI-based algorithms, earlier studies have shown specific ECG changes to be associated with PPCM, as well as adverse outcomes following diagnosis. These changes include sinus tachycardia, ST and T-wave abnormalities, QRS prolongation, QT prolongation, and left atrial abnormality.^36^, ^37^, ^38^, ^39^ However, these ECG characteristics have not been validated in large obstetric patient cohorts to verify its reliability and effectiveness for screening. Since the seminal work on AI-enabled ECG by Attia et al was published in 2019, several AI-ECG models have been developed for detecting LVSD, but few have been evaluated specifically in obstetric patient populations or among women of reproductive age. Other than studies performed by members of our team,^18^, ^19^, ^20^^,^^40^ 3 additional studies have been published, all focused specifically on detecting PPCM. A 2024 retrospective U.S. study evaluated an AI-ECG model (using 12-lead and single-lead ECGs), developed using a cohort of approximately 143,000 adult nonpregnant females with and without heart failure, for PPCM detection in 2 independent health systems (University of Tennessee Health Science Center, n = 115; and Atrium Health Wake Forest Baptist, n = 43). The model achieved AUROC values of 0.83 and 0.94 respectively for the 12-lead ECGs and 0.73 and 0.93 for lead-1 only ECGs.^31^ Notably in this study, the threshold value selected for binary classification of LVSD (LVEF <45%) was based on the smaller obstetric cohort (n = 158), as LVEF estimates were not available in the derivation heart failure cohort. Additionally, 2 small retrospective studies have been conducted among obstetric patients in South Korea (n = 157 and 204) published in 2022^29^ and 2023^41^ demonstrating AUROC values of 0.877 and 0.979 and PPV of 35% and 44%, respectively. In contrast to the prior studies, our current study includes a large obstetric patient cohort from diverse geographic regions. The cases detected were not limited to PPCM alone but included various forms of LVSD present during early pregnancy and up to 12 months postpartum. As such, this demonstrates its broad applicability for LVSD screening among women of reproductive age from preconception through pregnancy and the postpartum period. Although the high NPV in the hypothetical prevalence scenarios (Supplemental Table 2) is influenced, in part, by the low prevalence of LVSD, which is consistent with known estimates of PPCM in obstetric populations globally,^42^ NPV may be lower in higher prevalence scenarios,^43^^,^^44^ as such, our findings do not establish the AI-ECG as an independent diagnostic tool. From a clinical utility standpoint, the high NPV of the AI-ECG model supports its potential role as a first-line screening test to help rule out LVSD, prioritize echocardiography in higher risk patients, and complement established biomarkers (N-terminal pro b-type natriuretic peptide or B-Type Natriuretic Peptide) in risk-stratification pathways. Although AI-ECG has been shown to outperform natriuretic peptides for LVSD detection with minimal additional incremental benefit from combined testing in both obstetric and nonobstetric populations.^14^^,^^18^ However, the modest PPV highlights the need for confirmatory imaging after a positive result, due to potential false positive flags,^43^^,^^44^ and cautions against relying on the AI-ECG as a stand-alone diagnostic tool.

The primary strengths of this study include a large and diverse international patient sample, representing perhaps the largest external validation of the AI-ECG for detection of LVSD in an obstetric cohort. Additionally, the pragmatic nature of the study, which utilized LVEF results obtained at each participating site in accordance with local clinical and research protocols, reflects the practical application of this technology in real-world settings. Finally, the ability to compare 3 different 12-lead ECG devices provides valuable insights into model performance in various locations where similar devices may already be in use.

### Study Limitations

Study limitations include the following. 1) Limited availability of additional clinical variables, including cardiomyopathy type, for each patient included in the cohort, which precludes our ability to perform additional subgroup analyses or identify potential comorbid conditions that may influence AI model performance. 2) The use of predetermined model thresholds for detecting LVEF ≤40% that were not specifically calibrated as probabilities for LVSD for pregnant and postpartum females. This is particularly relevant given that PPCM is diagnosed at an LVEF value of <45%. 3) Echocardiographic images were not uniformly adjudicated across all sites, with LVEF values from EHR cohorts obtained from clinical reports rather than centralized image review, which may have contributed to measurement variability. 4) The prevalence of LVSD in our sample is likely higher than expected due to the requirement that patients have both an ECG and an echocardiogram, which, in clinical settings, could indicate the need for diagnostic testing due to clinical suspicion of underlying disease.

Nevertheless, this study demonstrates the effectiveness of the AI-ECG in a large cohort of obstetric patients, especially those with more severe forms of LVSD, characterized by a reduced ejection fraction (LVEF ≤40%) in accordance with the universal definition and classification of heart failure.^25^

## Conclusions

We demonstrate the effectiveness of the AI-ECG in detecting cardiomyopathy among obstetric patients across 11 health care institutions in 4 countries. The superior performance of the recently refined model, utilizing an expanded training dataset, underscores its potential as a promising and readily available screening tool for obstetric care globally. Prospective implementation studies will be an important next step to guide successful adoption of a universal AI-ECG screening program.

Perspectives **COMPETENCY IN MEDICAL KNOWLEDGE:** This international external validation study demonstrates that AI-ECG can identify LVSD in pregnant and postpartum women with strong discriminative performance across diverse geographic populations and clinical care settings.

**COMPETENCY IN SYSTEMS-BASED PRACTICE:** AI-ECG offers a simple and potentially scalable approach for identifying high-risk obstetric patients with LVSD, particularly in settings where access to echocardiography is limited. The high negative predictive suggests it could play a potential role in prioritizing diagnostic imaging, especially since ECGs are already embedded in existing screening and diagnostic care pathways.

**TRANSLATIONAL OUTLOOK:** Prior to broad adoption, prospective trials and implementation studies are needed to demonstrate sustained real-world effectiveness, assess clinical workflow integration, and clinical outcomes associated with AI-ECG screening in obstetric care. Further research is needed to define optimal model calibration, including determining optimal disease detection thresholds for pregnant and postpartum patients and evaluating performance across different ECG acquisition systems (standard and portable devices) to facilitate equitable implementation.

## Funding support and author disclosures

This study was partially supported by the Miami Heart Research Institute, Florida Heart Research Foundation, and through institutional funding at Mayo Clinic as well as various departments within the health care institutions (US and Israel) participating in the study. At the time of study initiation, Dr Adedinsewo received support from the Mayo Clinic Women’s Health Research Center and the Mayo Clinic Building Interdisciplinary Research Careers in Women’s Health (BIRCWH) Program funded by the National Institutes of Health (grant number K12AR084222). Additional support for this research came through Mayo Clinic’s Center for Clinical and Translational Science (UL1TR002377). Dr Griffin is supported by the National Center for Advancing Translational Sciences of the National Institutes of Health under Award Number UL1TR002378; the National Institute of Child Health and Human Development under Award Number U54HD113292; and the Health Resources and Services Administration under Award Number UR6MC50349. Miami Heart Research Institute had no role in the study design and conduct; data collection, management, analysis, and interpretation; manuscript preparation, review, or approval; and decision to submit the manuscript for publication. The content is solely the responsibility of the authors and does not necessarily represent the official views of the National Institutes of Health or the Health Resources and Services Administration. Anumana provided access to the AI-ECG models and provided technical expertise and support as needed prior to data analysis. Anumana did not participate in the data collection or analysis of the study data. Drs Attia and Friedman are co-inventors of several AI algorithms (including screening for low LVEF, QT tool, aortic stenosis, atrial fibrillation detection during normal sinus rhythm). These have been licensed to Anumana, AliveCor, and Eko. Mayo Clinic, Attia, and Friedman may benefit from their commercialization. Drs Noseworthy and Lopez-Jimenez and the Mayo Clinic have filed patents related to the application of AI to the ECG for diagnosis and risk stratification and have licensed several AI-ECG algorithms to Anumana. Dr Noseworthy and the Mayo Clinic are involved in potential equity/royalty relationship with AliveCor. Dr Noseworthy is a study investigator in an ablation trial sponsored by Medtronic; and has served on an expert advisory panel for Optum Labs. Drs Ranganathan and Awasthi are employees of Anumana, Inc. All other authors have reported that they have no relationships relevant to the contents of this paper to disclose.

## Supplementary material

Supplemental_Publication_License

## References

[1] Briller J., Trost S.L., Busacker A. (2024). Pregnancy-related mortality due to cardiovascular conditions. JACC Adv.

[2] Knight M., Bunch K., Tuffnell D. (2020). Saving Lives, Improving Mothers’ Care-Lessons learned to inform maternity care from the UK and Ireland Confidential Enquiries into Maternal Deaths and Morbidity 2016-18.

[3] Sliwa K., van der Meer P., Petrie M.C. (2021). Risk stratification and management of women with cardiomyopathy/heart failure planning pregnancy or presenting during/after pregnancy: a position statement from the Heart Failure Association of the European Society of Cardiology Study Group on Peripartum Cardiomyopathy. Eur J Heart Fail.

[4] Davis M.B., Arany Z., McNamara D.M., Goland S., Elkayam U. (2020). Peripartum cardiomyopathy: JACC state-of-the-art review. J Am Coll Cardiol.

[5] Karaye K.M., Ishaq N.A., Sa'idu H. (2020). Incidence, clinical characteristics, and risk factors of peripartum cardiomyopathy in Nigeria: results from the PEACE registry. ESC Heart Fail.

[6] Karaye K.M., Mohammed I.Y., Sa’idu H. (2024). Prevalence of left ventricular dysfunction and relationship with serum selenium in apparently healthy pregnant women: results from the PEACE registry. Global Cardiol.

[7] Bhatti S., Naz S., Gurmani S. (2024). Prospective Pakistan registry of echocardiographic screening in asymptomatic pregnant women. JACC Adv.

[8] Arany Z. (2024). Peripartum cardiomyopathy. N Engl J Med.

[9] Hameed Afshan B., Tarsa M., Graves Cornelia R., Grodzinsky A., Thiel De Bocanegra H., Wolfe D.S. (2024). Universal cardiovascular disease risk assessment in pregnancy. JACC Adv.

[10] ACOG (2019). ACOG practice bulletin no. 212: pregnancy and heart disease. Obstet Gynecol.

[11] Adedinsewo D., Morales-Lara A.C., Hardway H. (2024). Novel adaptation of the KCC-questionnaire for cardiomyopathy screening in a racially diverse obstetric population. NPJ Women's Health.

[12] Sliwa K., Petrie M.C., van der Meer P. (2020). Clinical presentation, management, and 6-month outcomes in women with peripartum cardiomyopathy: an ESC EORP registry. Eur Heart J.

[13] Yao X., Rushlow D.R., Inselman J.W. (2021). Artificial intelligence-enabled electrocardiograms for identification of patients with low ejection fraction: a pragmatic, randomized clinical trial. Nat Med.

[14] Adedinsewo D., Carter R.E., Attia Z. (2020). Artificial intelligence-enabled ECG algorithm to identify patients with left ventricular systolic dysfunction presenting to the emergency department with dyspnea. Circ Arrhythm Electrophysiol.

[15] Noseworthy P.A., Attia Z.I., Brewer L.C. (2020). Assessing and mitigating bias in medical artificial intelligence: the effects of race and ethnicity on a deep learning model for ECG analysis. Circ Arrhythm Electrophysiol.

[16] Harmon D.M., Carter R.E., Cohen-Shelly M. (2022). Real-world performance, long-term efficacy, and absence of bias in the artificial intelligence enhanced electrocardiogram to detect left ventricular systolic dysfunction. Eur Heart J Digit Health.

[17] Carter R.E., Johnson P.W., Strom J.B. (2026). Multisite, external validation of an AI-Enabled ECG algorithm for detection of low ejection fraction. JACC Adv.

[18] Adedinsewo D.A., Johnson P.W., Douglass E.J. (2021). Detecting cardiomyopathies in pregnancy and the postpartum period with an electrocardiogram-based deep learning model. Eur Heart J Digit Health.

[19] Adedinsewo D., Morales-Lara A.C., Hardway H. (2024). Artificial intelligence based screening for cardiomyopathy in an obstetric population: a pilot study. Cardiovasc Digit Health J.

[20] Adedinsewo D.A., Morales-Lara A.C., Afolabi B.B. (2024). Artificial intelligence guided screening for cardiomyopathies in an obstetric population: a pragmatic randomized clinical trial. Nat Med.

[21] K232699 low ejection fraction AI-ECG algorithm - cardiovascular machine learning-based notification software. https://www.accessdata.fda.gov/cdrh_docs/pdf23/K232699.pdf.

[22] Sounderajah V., Ashrafian H., Golub R.M. (2021). Developing a reporting guideline for artificial intelligence-centred diagnostic test accuracy studies: the STARD-AI protocol. BMJ Open.

[23] Sounderajah V., Guni A., Liu X. (2025). The STARD-AI reporting guideline for diagnostic accuracy studies using artificial intelligence. Nat Med.

[24] Cohen J.F., Korevaar D.A., Altman D.G. (2016). STARD 2015 guidelines for reporting diagnostic accuracy studies: explanation and elaboration. BMJ Open.

[25] Bozkurt B., Coats A.J., Tsutsui H. (2021). Universal definition and classification of heart failure: a report of the Heart Failure Society of America, Heart Failure Association of the European Society of Cardiology, Japanese Heart Failure Society and Writing Committee of the universal definition of heart failure. J Card Fail.

[26] Attia Z.I., Kapa S., Lopez-Jimenez F. (2019). Screening for cardiac contractile dysfunction using an artificial intelligence–enabled electrocardiogram. Nat Med.

[27] K250652 ECG-AI Low Ejection Fraction (LEF) 12-Lead algorithm - cardiovascular machine learning-based notification software. https://www.accessdata.fda.gov/cdrh_docs/pdf25/K250652.pdf.

[28] R Core Team (2019).

[29] Lee Y., Choi B., Lee M.S. (2022). An artificial intelligence electrocardiogram analysis for detecting cardiomyopathy in the peripartum period. Int J Cardiol.

[30] Kwon J.M., Kim K.H., Jeon K.H. (2019). Development and validation of deep-learning algorithm for electrocardiography-based heart failure identification. Korean Circ J.

[31] Karabayir I., Wilkie G., Celik T. (2024). Development and validation of an electrocardiographic artificial intelligence model for detection of peripartum cardiomyopathy. Am J Obstet Gynecol MFM.

[32] Parsa S., Keyes T., Dash D. (2026). Designing clinically useful AI: a blueprint for impact. NEJM AI.

[33] Collet J.P., Thiele H., Barbato E. (2021). 2020 ESC guidelines for the management of acute coronary syndromes in patients presenting without persistent ST-segment elevation. Eur Heart J.

[34] Kontos M.C., de Lemos J.A., Deitelzweig S.B. (2022). 2022 ACC expert consensus decision pathway on the evaluation and disposition of acute chest pain in the emergency department: a report of the American College of Cardiology Solution Set Oversight Committee. J Am Coll Cardiol.

[35] Le Gal G., Robert-Ebadi H., Thiruganasambandamoorthy V. (2026). Age-adjusted D-Dimer cutoff levels to rule out deep vein thrombosis. JAMA.

[36] Karaye K.M., Lindmark K., Henein M.Y. (2016). Electrocardiographic predictors of peripartum cardiomyopathy. Cardiovasc J Afr.

[37] Hoevelmann J., Viljoen C.A., Manning K. (2019). The prognostic significance of the 12-lead ECG in peripartum cardiomyopathy. Int J Cardiol.

[38] Honigberg M.C., Elkayam U., Rajagopalan N. (2019). Electrocardiographic findings in peripartum cardiomyopathy. Clin Cardiol.

[39] Sliwa K., Hilfiker-Kleiner D., Damasceno A. (2025). Peripartum cardiomyopathy. Lancet.

[40] Kinaszczuk A., Morales-Lara A.C., Garzon-Siatoya W.T. (2025). Artificial intelligence tools for preconception cardiomyopathy screening among women of reproductive age. Ann Fam Med.

[41] Jung Y.M., Kang S., Son J.M. (2023). Electrocardiogram-based deep learning model to screen peripartum cardiomyopathy. Am J Obstet Gynecol MFM.

[42] Adedinsewo D.A., Brown H.L., Hameed A.B. (2026). Heart failure occurring in the perinatal period: a scientific statement from the American Heart Association. Circulation.

[43] Cosby K., Yang D., Fineberg H.V. (2024). Assessing diagnostic performance. NEJM Evid.

[44] Klontzas M.E., Groot Lipman K.B.W., Akinci D. (2026). ESR essentials: common performance metrics in AI-practice recommendations by the European Society of Medical Imaging Informatics. Eur Radiol.

