# Supplementary material for: Validating ECG-Based Artificial Intelligence Models for Cardiomyopathy Detection in Obstetric Patients: A Multisite International Study
Source: JACC Adv. 2026 Sep 22;5(11):103321. doi: 10.1016/j.jacadv.2026.103321 (PMC13682377; doi:10.1016/j.jacadv.2026.103321)
Supplement: Supplementary file 2 [file mmc2.docx]

SUPPLEMENTAL APPENDIX

**DESCRIPTION OF PARTICIPATING COHORTS**

**United States (Florida, Georgia, and North Dakota)**

Data from the United States (University of Florida Jacksonville, Grady Memorial Hospital/Morehouse School of Medicine, and Essentia Health Heart and Vascular Center, Fargo) were derived from healthcare institutions where ECGs and echocardiograms were obtained as part of routine clinical care. As a result, the study population were likely higher-risk individuals undergoing cardiovascular evaluation, including those with symptoms, abnormal cardiac findings, or suspected cardiovascular disease, rather than representing an unselected obstetric screening population.

**Israel (Sheba Medical Center)**

The Israeli cohort was obtained from a large tertiary care center and included pregnant and postpartum patients with available paired ECG and echocardiographic data obtained as part of routine clinical care. Similar to the U.S. sites, data were derived from clinically acquired studies and therefore likely reflect a patient population undergoing cardiovascular assessment rather than universal screening.

**Nigeria (SPEC-AI Trial Sites)**

Data from Nigeria were obtained from participants in the intervention arm of the SPEC-AI randomized clinical trial who had a resting ECG and an echocardiogram done at baseline. The SPEC-AI trial enrolled pregnant and postpartum women from six tertiary healthcare institutions in Nigeria. Recruitment was not restricted to individuals with known or suspected cardiovascular disease, as such the cohort included both symptomatic and asymptomatic women and may provide a more pragmatic obstetric screening population in Nigeria.

**Pakistan (PRESAP Registry)**

The Pakistan cohort was derived from the Pakistan Registry of Echocardiographic Screening in Asymptomatic Pregnant Women (PRESAP), a prospective screening registry of pregnant women without a prior diagnosis of cardiovascular disease. The PRESAP study enrolled asymptomatic pregnant women and most closely approximates a population-based obstetric screening setting in Pakistan.

**SUPPLEMENTAL TABLES**

**Supplemental Table 1.** Overall diagnostic performance metrics for the AI-ECG models in detecting LVEF < 45%

| **Measure** | **v2.5.0** | **v3.0.0** |
| --- | --- | --- |
| Prevalence | 7.8% (6.6%, 9.2%) (138/1,763) | 7.8% (6.6%, 9.2%) (138/1,763) |
| Sensitivity | 43.5% (35.1%, 52.2%) (60/138) | 60.9% (52.2%, 69.1%) (84/138) |
| Specificity | 96.9% (95.9%, 97.7%) (1,574/1,625) | 96.7% (95.7%, 97.5%) (1,571/1,625) |
| AUROC | 0.852 (0.817, 0.887) | 0.909 (0.880, 0.938) |
| Positive Predictive Value | 54.1% (44.3%, 63.6%) (60/111) | 60.9% (52.2%, 69.1%) (84/138) |
| Negative Predictive Value | 95.3% (94.1%, 96.3%) (1,574/1,652) | 96.7% (95.7%, 97.5%) (1,571/1,625) |

 **Abbreviations:** AUROC, Area under the receiver operating characteristic curve.

**Supplemental Table 2.** Expected AI-ECG model performance at various hypothetical screening prevalence rates

| **Model** | **Prevalence** | **PPV** | **NPV** | **FP per 1000** | **FN per 1000** |
| --- | --- | --- | --- | --- | --- |
| v2.5.0 | 2.00% | 22.8% (17.6%, 29.1%) | 98.9% (98.7%, 99.0%) | 31 | 11 |
|  | 1.00% | 12.8% (9.6%, 16.9%) | 99.4% (99.3%, 99.5%) | 31 | 5.4 |
|  | 0.50% | 6.8% (5.0%, 9.2%) | 99.7% (99.7%, 99.8%) | 32 | 2.7 |
|  | 0.25% | 3.5% (2.6%, 4.8%) | 99.9% (99.8%, 99.9%) | 32 | 1.3 |
|  | 0.10% | 1.4% (1.0%, 2.0%) | 99.9% (99.9%, 100.0%) | 32 | 0.5 |
|  | 0.05% | 0.7% (0.5%, 1.0%) | 100.0% (100.0%, 100.0%) | 32 | 0.3 |
| v3.0.0 | 2.00% | 27.6% (22.2%, 33.7%) | 99.2% (99.1%, 99.4%) | 34 | 7.2 |
|  | 1.00% | 15.9% (12.4%, 20.1%) | 99.6% (99.5%, 99.7%) | 34 | 3.6 |
|  | 0.50% | 8.6% (6.6%, 11.1%) | 99.8% (99.8%, 99.9%) | 34 | 1.8 |
|  | 0.25% | 4.5% (3.4%, 5.9%) | 99.9% (99.9%, 99.9%) | 34 | 0.9 |
|  | 0.10% | 1.8% (1.4%, 2.4%) | 100.0% (100.0%, 100.0%) | 34 | 0.4 |
|  | 0.05% | 0.9% (0.7%, 1.2%) | 100.0% (100.0%, 100.0%) | 34 | 0.2 |

**Abbreviations:** PPV, positive predictive value; NPV, negative predictive value; FP, false positive; FN, false negative

**Supplemental Table 3.** Sensitivity Analysis: AI-ECG model performance excluding data from Pakistan

| **Measure** | **v2.5.0** | **v3.0.0** |
| --- | --- | --- |
| Prevalence | 7.2% (5.9%, 8.6%) (110/1,536) | 7.2% (5.9%, 8.6%) (110/1,536) |
| Sensitivity | 50.0% (40.3%, 59.7%) (55/110) | 70.9% (61.5%, 79.2%) (78/110) |
| Specificity | 96.4% (95.3%, 97.3%) (1,375/1,426) | 96.1% (94.9%, 97.0%) (1,370/1,426) |
| AUROC | 0.883 (0.852, 0.915) | 0.937 (0.913, 0.961) |
| Positive Predictive Value | 51.9% (42.0%, 61.7%) (55/106) | 58.2% (49.4%, 66.7%) (78/134) |
| Negative Predictive Value | 96.2% (95.0%, 97.1%) (1,375/1,430) | 97.7% (96.8%, 98.4%) (1,370/1,402) |

**Abbreviations:** AUROC, Area under the receiver operating characteristic curve
